# Single mothering as experienced by Burundian refugees in Australia: a qualitative inquiry

**DOI:** 10.1186/s12912-017-0260-0

**Published:** 2017-11-25

**Authors:** Lily P. Tsai, Jennieffer A. Barr, Anthony Welch

**Affiliations:** School of Nursing, Midwifery, and Social Sciences, Central Queensland University, 160 Ann Street, Brisbane, Queensland 4000 Australia

**Keywords:** Refugees, Single mothering, Qualitative research, Acculturation, Parenting strategies

## Abstract

**Background:**

Refugee mothers have fled from their homeland to escape persecutions with their children only to find other threats to their well-being in the new country. Building on previous research, it is known that being a new immigrant is challenging and requires adaptation. The adaptation process, known as acculturation, may not be successful leading to psychological distress. It is also known that a generation gap can occur when children acculturate faster than their parents. What was lacking was understanding about the experiences of single refugee mothers.

**Methods:**

Interpretative phenomenological study was undertaken to explore the lived experiences of eight Burundian refugee single mothers in Australia. Data were collected by in-depth interviews. Each interviews were transcribed and analyzed using thematic analysis.

**Results:**

Findings revealed three themes. First theme ‘Traditional mothering practices of Burundian culture’ illustrated mothering strategies as practiced prior to their arrival in Australia including mothering with sufficient social support, strong position of parents, and regular use of physical disciplining. Second theme ‘Challenges identified after arrival to new country’ revealed that mothers felt their children acculturated faster than themselves which led to intergenerational gap. This has also led participants to live in a continuous dilemma, experiencing inner conflicts and struggles associated with their mothering practices, especially when mothers had arrived with a lack of knowledge relating to acceptable mothering practices in a new culture. Final theme, ‘Reforming family life in Australia’ highlighted the decisions made by single refugee mothers which is to embrace both new and original cultures, leading to successful acculturation. However, lack of appropriate knowledge of acceptable mothering practices led to involvement of legal authorities who threatening to remove children from the mother’s care. This has led mothers feeling change of power from ‘mother to child, ‘to child to mother’, raises concerns for family wellbeing.

**Conclusions:**

A need for parenting information when entering a new country including education about any legal obligations for parents such as a Child Protection Act will assist successful acculturation. As nurses are likely to encounter refugee single mothers, they are well placed to provide support and education to new refugee single mothers.

## Background

Nurses aim to provide culturally sensitive practice to enhance wellbeing. Whilst nurses already have well-established evidence of guiding practice for refugee and immigrant parents, less is known about mothering practices of single refugee mothers. This was the impetus for undertaking the research reported in this paper.

Nurses are more likely to encounter single refugee mothers than other healthcare professionals, particularly those who work in primary health settings. Hence, nurses need to be open to the health and social challenges faced by mothers in relation to their mothering practices. However, nurses may not always have well-developed cultural understandings of the new and emerging groups entering the country.

Globally, more than 65 million people are forced to leave their motherland. Of these 21 million persons become refugees, and more than half are women and girls [1]. As one of top three preferred resettlement countries, the Australian Government allocates exceptional visa to women and girls who are at risk of violence [2].

The process of adaptation to a new environment, known as acculturation, is a challenging process of learning, transforming, and changing for any new settlers [3]. Previous literature has examined the multiple layers of acculturation as experienced by mothers. The first layer is acculturation of mothers themselves. Evidence highlighted that mothers experience challenges with competing norms, values, and beliefs of original society and of the new society. Therefore, for example, successful adaptation involves a person to learn the language and lifestyle of the new environment [4]. In addition, another strategy leading to successful acculturation is to establish social and cultural networks in a new country [5]. This will be important particularly for those mothers who embrace the upbringing of children with social and familial support in the form of extended families and communities.

Acculturation also influences mothering practices. Frist, maternal ethnicity, length of stay in a new country, and successfulness of acculturation influence mothering practices [3]. Moorhouse and Cunningham [6] argued that as acculturation progresses, maternal ability to facilitate the development of children also strengthens. Second, acculturation impacts on family dynamics through different paces of acculturation as experienced by each family members [7].

However, acculturation can negatively impact on the health and wellbeing of refugee mothers. Lui and Rollock [8] argues acculturation is associated with psychological distress, claiming that unsuccessful acculturation and the development of depression is evident. Of concern is the findings from the study by Kira et al. [9] who found that immigrants are 60% less likely to be diagnosed with psychological distress and associated health issues, yet be suffering from mental health issues. Similarly, Springer et al. [10] identified that Somali Bantu refugees in Canada are likely to deny their mental health concerns while healthcare providers identified their stress reactions. The knowledge of psychological distress and health issues for refugees is important. However, there is little known about such distress for mothers who are refugees. At the time of this study, no paper was published about Burundian mothers who came as refugee with single mother status.

Single status of a mother is an additional factor which influences the wellbeing of refugee mothers. Borrows et al. [11] argues that single mothers are more vulnerable than married or partnered mothers, in terms of living a life of poverty and reduced health and wellbeing. Furthermore, single mothers are twice more likely to experience high levels of perceived stress than partnered mothers, and therefore, single mothers are more likely to experience depression [12].

Steward et al. [13] highlighted that refugee women have three-fold increased risk of developing postnatal depression in comparison to women born in the country. Of concern is the conclusion by Copeland and Harbaugh [14] that African families led by single mothers had increased level of acculturation stress and subsequent mental distress than any other ethnic groups of single mothers or partnered families.

It is now well established from many studies that language proficiency of a new country influences the acculturation of mothers [15]. Low language proficiency of refugee mothers may lead to confusion, anxiety, limited knowledge about accessing healthcare services, and may prevent their interaction with the community [4]. Nurses will be challenged to provide effective care when mothers do not speak the same language [16]. Whilst research has contributed to a body of knowledge about immigrant and refugee mothers living in a new country, there are still gaps in understanding in this topic area. In particular, there is less known about single refugee mothers. Of particular concern is a dearth of knowledge about the lived experiences of single mothers who are refugees. Such understandings are important in providing person-centered care.

### Purpose and the aim of the study

The purpose of this study was to increase understanding of single mothers’ experiences of acculturation and mothering practices as a refugee. This study was conducted as part of a larger study for the fulfillment of Master’s degree. At the time of this study, there was no research which has explored the experiences of single Burundian mothers who have fled their homeland and in search of a safe environment for themselves and their children.

## Method

### Methodology

Interpretive hermenutic phenomenological methodology guided by Heidegger [17] was implemented to access Burundian mothers’ lived experiences about mothering in a new country. Phenomenology is chosen for researchers to access the meaning of life experiences of others [18]. Furthermore, to make sense of these experiences, hermeneutical phenomenology uses lived experience as a tool for developing understanding of the social, cultural, political and historical context in which those experiences occur [18]. Therefore, the research question asked ‘what does it mean to Burundian mothers to be a single mother in Australia?’ The main objective was to explore mothering as experienced by single Burundian refugee mothers in Australia, using a qualitative interview technique. For this study, single mother was defined as a mother who has no spouse or partner in the household but who lived with at least one child.

### Recruitment process

The participant group was drawn from a city in Australia where these women typically live (location not noted to ensure anonymity, as this population in Australia is small in numbers). The subsequent sample included those who could provide rich details about single mothering in a new culture, and who were Burundian refugee single mothers. Of the fifteen potential participants, seven single refugee mothers agreed to participate. In addition, snowball technique was also implemented by asking those mothers already participating if they know any other mothers in their social networks who might be interested in being involved in the research [17]. This resulted in an additional participant. Therefore, in total, eight single refugee mothers participated in this study.

No official interpreters were available but one participant was asked to interpret during the other interviews (data collection). She was fluent in both English and Burundian language. Participants consented to the interpreter being present. Therefore, all communication with participants were conducted by a researcher and the interpreter.

The identified single mothers were contacted by phone with an invitation to participate in the study. The initial phone call included a description of the objectives of the study, the method of the project, assurances of anonymity and confidentiality, and directions regarding how to participate in the study. After the study was explained in detail and the individual agreed to participate, a researcher traveled to a location of participant’s choice that assured privacy and confidentiality. Locations included participant’s home and an office room in a community service.

### Ethical consideration

The ethical clearance was granted from a tertiary education institution’s Human Research Ethics Committee. All participants were given a copy of the informed consent form to review and sign. Any questions that the participant had were addressed prior to proceeding with the interview. All participants gave written consent for participation after receiving written and oral information about the study. No time limit had been set for the interview, however, in average, interviews were completed in two hours.

### Participants

Eight single refugee mothers participated in this study. The demographic data are summarized in Table 1. The age of participants ranged from 20 to 55 years. All participants arrived in Australia prior to 2008; thus, they had been living in Australia for two to six years at the time of the data collection. The number of children of all participants ranged from one to eight. The ages of the oldest child in each family ranged from 2 to 27 years of age.

**Table 1:** Summary of demographics of participants

	Median (range)
Age (years old)	39 (20–55)
Number of years in Australia (years)	3 (2–6)
Number of children	5 (1–8)
Age of first child (years old)	12 (2–28)

### Data collection

Each woman participated in an audio recorded interview that focused on her experience of becoming a Burundian single mother with refugee status in Australia. Clarifying questions were used to expand the woman’s story as needed. The interviews were then transcribed into written material after each interview and reviewed for thematic content. At this time, a pseudonym was given to each participant. Two researchers independently analyzed the data to ensure intercoder reliability. One of the researchers did not perform the interviews, and therefore, had never met the participants. Transcripts were manually coded to identify recurring themes that reflected common meanings and shared phenomena.

### Data analysis

Following Benner’s [19] analytic approach for hermeneutic phenomenology, data analysis of this study used three interrelated processes which were the search for paradigm cases, thematic analysis, and analysis of exemplars. Identifying paradigm cases is a strategy for gaining understanding of ‘ways of being in the world’ [19]. Thematic analysis allow researchers to compare and contrast similarities across cases. Exemplars are used to illuminate aspects of paradigm cases or theme [19]. The responses to the open-ended questions were thematically analyzed and examined for emerging patterns or themes. Analysis was performed by reading and rereading each interview to identify relationships between themes. As well as commonly shared themes, attention was given to unique experiences.

## Results

Three major themes emerged from the study: *Traditional mothering practices of Burundian culture*, *Challenges identified after arrival to a new country*, and *Need of reframing family life in a new country*.

### Traditional mothering practices of Burundian culture

Three mothering practices were highlighted by participants as traditional mothering practices of Burundian culture. First, traditionally, Burundian culture raise children as a community, which is also known as ‘village mothering’. The term ‘village mothering’ is where the community assumes collegial responsibility for the development of a child into adulthood. Therefore, mothers had a strong relationship and connection with members in the village. Robyn illustrated her connection with other families in her traditional community when she said:

*I loved Burundi when the whole family lived close by and I knew everyone around me. When we had problems in our family, we had the whole group supported us in problem-solving our way through the issues.*

Naomi also spoke of the bond she had with her community in stating:

*I knew every single person in my community. We had a strong bond. When I needed something, all I had to do was open the door and ask. If I had issues I knew I could count on my local community for support and at times direction.*

Second, traditional mothering practices of Burundian culture were also characterized by their style of parenting. The position of parents was one of absolute authority. Children are expected to be obedient, to listen, and show respect to all, especially to the elderly in the community. For example, Luna explained:

*I expect my children to listen to me as I’m their mother. I expect them to behave with respect, honor my position within the family, and always listen to me*.

Kay concluded:

*I expect them to obey me completely, without questioning*.

Third, participants also highlighted that to maintain their authority, use of physical discipline was socially sanctioned by the Burundian community. Naomi stated that:

*When my child did something bad, I could punish them in any way I wanted to*.

Luna explained that such an approach to disciplining children was engraved in their daily practices which were never questioned by the Burundian community.

*We didn’t have anyone to report it. We as the parents were the authority with the support and sanction of our community.*

Yuli explained further:

*As a mother, you have the right to teach your children when they misbehave. Disciplining my children involves physical punishment but only in extreme situations. In reality, it is only a smack or two, nothing too physical.*

### Challenges identified after arrival to a new country

After participants arrived at a new country, refugee single mothers found themselves in situations where their traditional mothering practices were challenged by a different set of social expectations of the new country. This was particularly evident in relation concerning the place of authority of the mother and the use of physical discipline; leading to a feeling of the intergenerational gap with their children. Relocating from a culture in which the parents and community had the overarching authority about the way in which children should be raised with the rights of the child of secondary concern was in stark contrast to the Australian culture in which the rights and freedoms of the child are of fundamental importance.

First, requiring to change to a new cultural norms of parenting was described by all participants as causing major conflict between them and their children. Such a contrast in cultural values and expectations became a source of mother-child conflict. The participants spoke of the insidious change in the way their children began to challenge their authority and assert their own rights. As stated by Ellen:

*I was stunned when my son told me that he has the right to choose what he wanted to do or listen to. He had never spoken like this before. I felt he’s out of control and I didn’t know what to do.*

Refugee single mothers were confused and lacked knowledge and skills about appropriate parenting as according to Australia laws about physical discipline. Physical discipline is unlawful in this country and the Government officials (the Department of Communities, Child Safety, and Disability Services) can withdraw children from a mother’s care if ongoing use of physical discipline is used. At the time of this study, all participants had experienced or witnessed children being removed and/or the police or other officials threatening to remove children from their custody. As Naomi, described:

*Gradually the behavior of my eldest child changed to a point that when I tried to discipline him, because of his poor behavior, he threatened me with telling the police that I was abusing him. I was frightened and did not know what to do. I had never been in such a situation. I had to back down.*

Luna shared a similar experience in stating:

*When I wanted to discipline my children, they started to run to the phone, threatening me by pretending they are going to call the police. I could not believe that my children would ever do this. I began to question where did I go wrong?*

The exhibition of such behaviors by some of the participant’ children left the participants with an overwhelming sense of disillusionment and failure. Robyn concluded “I wasn’t used to living like this. I felt that I had one less thing I had to do for my children.”

Second, the emergence of a growing disparity between mother and child expectations of what constitutes mothering practices led to an increasing divide, further expanding the intergenerational gap. Participants spoke of their increasing fear of losing their children to the Australian culture as shared by Cosmos:

*When we first arrived we were very close as a family. The children depended on me for all their needs. As time passed and the children became more confident, I began to feel that I was less needed as if I was gradually losing them.*

Luna shared similar feeling:

*Despite all my efforts to remain a close family, I began to feel a growing sense of distance between my children and myself. It seemed I was becoming irrelevant in their lives.*

Naomi expressed similar thoughts to those of Luna stating:

*As my children became more and more part of the Australian culture I felt there was a growing gap between us. I struggled to understand them as I once did. Communicating with them became increasingly difficult.*

Kay was more direct in talking about the growing gap between herself and her children:

*It started with the language. As my children became more capable of speaking English that was when it first started as I had little understanding of what they were talking about. It was as if we were in different worlds.*

Many of their cherished values of mothering were brought into question as a number of participants began to evaluate what they needed to do to change the situation. Reframing family life seemed to be the only plausible answer.

### Reframing family life in Australia

Mothering in a new country was a difficult process for refugee single mothers. This process involved letting go of some cherished values about parenting and attempting to make the cultural transition to a different value system and set of cultural norms. Initially, participants spoke of being lost as to where to start and felt that they were now in unchartered waters. Kay shared:

*I felt I had to stop what was happening to my family but did not know where to begin. The only place I found comfortable with was simply trying to talk with them which was difficult at first as they did not want to listen.*

For Kay, it was ‘quiet persistence’ that changed her situation as she consistently applied new child discipline strategies. For Naomi, it was more about ‘setting new guidelines about behaving appropriately.’ Luna attempted to focus on acknowledging that her children had rights and that having such rights also brought with it responsibilities:

*It was no use in trying to take away their rights that would have only ended up in further conflict. What I had to do was talk about what having a different set of rights to those of our culture meant and how they needed to behave.*

At the time of this research, participants were still struggling with the need to reframe what it meant to be a mother in a new cultural context on a daily basis.

## Discussion

There are few studies that have investigated acculturation as experienced by refugee single mothers. Therefore, this study aimed to increase knowledge by exploring and understanding experiences of refugee single mothering in a new country. This study confirmed and supported two established claims currently found in the literature in relation to acculturation. This study found that single refugee mothers decided to embrace both the new and original cultures. Such decision of embracing both cultures involves the least level of stress [20]. Second, like previous research, mothers also reported their children acculturated at a faster pace than they did which led them to experience intergenerational gap. This intergenerational gap meant that participants had to make decisions about which original cultural values and identities they retain, while simultaneously taking on the cultural values of the host country. For example, a difficulty for Burundian single mothers was learning new mothering and disciplinary skills, as previous strategies are no longer appropriate in Australia.

Like previous studies (i.e. [4, 10, 21, 22]), all participants in this study reported problems with communication. Schweitzer et al. [22] found that 70% of Burmese refugees reported limited language proficiency of the new country is a serious issue. One significant consequence of low language proficiency of a new country is low rates of access to mental health services [4].

Additionally, refugees need to navigate through healthcare services in a new country [10, 21, 23]. Many participants also reported this as a challenge. People who are refugees require services that respond effectively to their mental health needs [21, 22]. Participants of this study also expressed that there are limited locations where they can seek help. Therefore, culturally appropriate social and health services play an important role in assisting refugees to achieve successful resettlement.

Refugee single mothers are living in a continuous dilemma, experiencing inner conflict and struggles associated with mothering practices [21]. To overcome acculturation challenges, refugee mothers need to empower themselves, acknowledging tradition, and become transformative in the new culture. The participants of this study showed signs of achieving some transformation but continued to be challenged by other aspects of the cultural conflict.

Relocation can create a sense of isolation, confusion, frustration, and disempowerment [2]. The knowledge of the new culture with culturally specific information is a predictive factor of successful acculturation. For example, participants of this study expressed some fear of losing their children due to a lack of awareness and understanding about the Child Protection Act in Australia [24]. Most participants had received threats from authorities signaling that they could lose the custody of their children, while some participants actually had their children removed from their custody as a result of the use of traditional discipline strategy (physical disciplining).

New significant findings related to being a single mother without support of a partner is useful new knowledge as many refugees from war-torn countries may be single mothers. The participants in this study expressed how challenging it was to discipline their children as a single mother and without using physical discipline. Participants admitted that they did not know alternative ways to discipline their children. Many mothers acknowledged that their children threatened to call the emergency number if mothers did not permit them to engage in the child’s preferred activities. This change of power from ‘mother to child’, ‘to child to mother’, described by these women raises concerns for family wellbeing.

There were two limitations of this study. First, this was a qualitative study which cannot to be generalized to the broader population. However, the results may be applicable to other refugees and other countries. Second, this study involved a gatekeeper who provided access to potential participants to the study. This may have led to participant selection bias. However, to address this potential bias, snowball sampling was used where the participants themselves were asked if they knew other mothers who may be interested in trying to minimize this limitation.

## Conclusion

A significant finding was the need to change parenting strategies when moving to a new country. Learning socially acceptable child disciplinary strategies in a new country was not easy for participants and at times potentially detrimental to the family dynamics. Refugee children learnt to manipulate mothers when involved with authorities such as the police. The problem develops when refugee single mothers are not able to communicate in the same language. This problem meant the mothers were unable to preempt and address the problem before legal groups, like police, become involved.

As Jones et al. [25] argued, this study supports the claim that former parental authority is compromised as children developed a higher level of language proficiency. A consequence of this change is family disharmony and an inability by mothers to discipline children at times. Education and support in parenting is likely to facilitate successful adaptation.

Nurses will play a significant role in facilitating acculturation as they are likely to encounter refugee single mothers. Nurses can assist these mothers by suggesting alternative child discipline strategies. In addition, nurses are also to help refugee single mothers to navigate and locate health care systems. For example, parenting classes and mother groups aiming to assist refugee single mothers to gain skills in navigating new culture. School nurses can play a part by facilitating partnerships with refugee families and working with children. By teaching the children the consequences of being removed from their mother’s care children may be less likely to threaten to call the police for unnecessary and non-violent situations. Finally, nurses have an obligation to assess mental health and facilitate further access to specialist culturally appropriate services to enhance psychosocial wellbeing.

It is recommended that further research is conducted for refugees. Research needs to seek understanding of ethnic cultures and identify specific challenges and strategies to facilitate successful acculturation. Particular focus should be given to minority groups such as single mothers who face additional challenges when compared to two parent families. In addition, family intervention should be instigated to facilitate dialogue between mothers and children. This requires ongoing small group discussions to provide opportunities for both mother and child to share issues and challenges in a safe and open environment.

## Abbreviation

UNHCR: United National High Commissioner for Refugees
